# PEARL: novel protein targets recommendation model via heterogeneous graph representation learning

**DOI:** 10.1093/bib/bbag551

**Published:** 2026-10-08

**Authors:** Zixuan Liu, Jiasi Luan, Zhen Zhang, Kui Tang, Xingyu Qin, Zhixiao Qi, Borui Zhang, Jun Liao

**Affiliations:** School of Artificial Intelligence, China Pharmaceutical University, 639 Longmian Avenue, Jiangning District, Nanjing 211198, Jiangsu, China; School of Artificial Intelligence, China Pharmaceutical University, 639 Longmian Avenue, Jiangning District, Nanjing 211198, Jiangsu, China; School of Artificial Intelligence, China Pharmaceutical University, 639 Longmian Avenue, Jiangning District, Nanjing 211198, Jiangsu, China; School of Artificial Intelligence, China Pharmaceutical University, 639 Longmian Avenue, Jiangning District, Nanjing 211198, Jiangsu, China; New Drug Screening and Pharmacodynamics Evaluation Center, State Key Laboratory of Natural Medicines, China Pharmaceutical University, 639 Longmian Avenue, Jiangning District, Nanjing 210009, China; School of Artificial Intelligence, China Pharmaceutical University, 639 Longmian Avenue, Jiangning District, Nanjing 211198, Jiangsu, China; School of Artificial Intelligence, China Pharmaceutical University, 639 Longmian Avenue, Jiangning District, Nanjing 211198, Jiangsu, China; School of Artificial Intelligence, China Pharmaceutical University, 639 Longmian Avenue, Jiangning District, Nanjing 211198, Jiangsu, China

**Keywords:** recommendation system, heterogeneous graph network, multimodal feature integration, peptide drug repurposing, peptide–protein interaction prediction

## Abstract

Peptide drug repurposing depends on identifying likely protein targets for a query peptide across a large candidate space, yet most existing peptide–protein interaction models are optimized for isolated pairwise classification rather than target prioritization. Here, we present PEARL, a recommendation-oriented heterogeneous graph learning framework for peptide target recommendation. PEARL represents peptides and proteins as distinct node types connected by known peptide-protein associations and multi-view peptide similarity, and combines graph-based representation learning with similarity-aware score refinement to rank candidate targets for a query peptide. On the UMPPI benchmark, PEARL achieved mean area under the receiver operating characteristic curve (AUC), area under the precision–recall curve (AUPR), and Matthews correlation coefficient values of 0.868, 0.855, and 0.571 in five-fold cross-validation, outperforming the strongest baseline, HGT-PepPI, by 4.4, 4.8, and 7.0 percentage points, respectively. Under clustered cold-start evaluation, PEARL delivered the best overall AUC across the novel-protein, novel-peptide, and novel-pair settings, indicating robust generalization under increasing sequence dissimilarity. PEARL also remained the top performer on two independent test sets, reaching an AUC of 0.863 on Test167 and 0.871 on Test1440. Additional analyses showed that PEARL can be effectively calibrated and that its learned latent space groups functionally related targets beyond simple sequence identity. In a case study of the unseen therapeutic peptide CIGB-814, PEARL ranked SPSB2 as the top candidate target. Docking and molecular dynamics simulations supported a stable binding mode, and surface plasmon resonance further confirmed direct binding with an apparent KD of 5.538 μM. Together, these results support PEARL as a scalable computational framework for peptide target recommendation and peptide drug repurposing.

## Introduction

Over the past two decades, peptides have gained considerable interest as therapeutic agents due to their high specificity, low toxicity, and adaptability, enabling their broad applications in medicine and biotechnology, with a growing number of peptide-based therapeutics entering clinical use [1–5]. Despite their enormous potential, the successful development of peptide therapeutics depends strongly on the identification of appropriate targets and therapeutic indications. As a promising solution, drug repurposing offers a more efficient and cost-effective pathway by identifying new therapeutic indications for existing approved drugs [6, 7]. For peptide therapeutics, identifying new indications often amounts to discovering novel protein targets that can interact with them, a process that requires extensive reverse screening. Given that up to 40% of all protein–protein interactions (PPIs) are mediated by short peptide motifs [8], the strategic identification of potential therapeutic applications for peptides depends critically on accurate prediction of peptide–protein interactions (PepPIs) [9, 10]. However, the experimental determination of PepPIs is both resource-intensive and time-consuming, a challenge compounded by the often transient and flexible nature of peptide-protein complexes [10, 11].

Recent advances in deep learning, together with the rapid accumulation of biological data, have greatly accelerated the development of computational methods for PepPI prediction [12]. Existing approaches mainly rely on graph convolutional networks (GCNs), convolutional neural networks (CNNs), and Transformer-based architectures. For example, InterPepRank [13] employs GCNs to improve the prediction of peptide binding conformations and binding sites, while Struct2Graph [14] uses graph-based mutual attention to infer PPIs from structural information. Convolutional Attention-based Neural Network for Multi-level Peptide–Protein Interaction Prediction (CAMP) [15] adopts a CNN-based framework to integrate peptide and protein sequence features and identify peptide binding residues. PepNN [16] introduces a Transformer-based reciprocal attention mechanism for peptide binding site prediction, and fine-tuned AlphaFold2 [17] has also been explored to improve PepPI prediction using existing binding data. Although these methods have demonstrated promising performance, most were developed to determine whether a single peptide-protein pair interacts, rather than to prioritize actionable protein targets for a peptide across a large candidate space.

Despite recent advances in PepPI prediction, existing methods remain suboptimal for peptide drug repurposing. This limitation arises largely because peptide repurposing is inherently a proteome-scale target identification problem, whereas most available approaches are designed for pairwise interaction prediction. This mismatch leaves several key challenges insufficiently addressed. First, most current methods are optimized for binary classification rather than for prioritizing a small number of plausible targets from a large candidate pool, which is the practical objective of peptide repositioning. Second, current approaches typically treat PepPIs as isolated events, with limited ability to capture global dependency patterns across the broader peptide-protein association landscape. In practice, known interactions involving related peptides may provide informative context for identifying additional targets, yet such higher-order regularities are rarely exploited in a systematic manner. Third, peptides and proteins are biologically distinct entities, but many existing frameworks do not explicitly model this heterogeneity in a way that supports relation-aware target inference at scale. Together, these limitations indicate that accurate pairwise PepPI prediction alone is insufficient for peptide repurposing, and that the field requires models explicitly designed for large-scale target recommendation.

To address these challenges, we propose PEARL, a unified framework for protein target recommendation in peptide repositioning. PEARL reformulates PepPI prediction as a heterogeneous graph learning problem in which peptides and proteins are represented as distinct node types and connected through known peptide-protein associations together with peptide similarity structure, while allowing additional target-side relational information when informative. Beyond relation-aware representation learning, PEARL further incorporates a recommendation-oriented ranking strategy that propagates evidence from related peptides, thereby aligning the model with the practical objective of large-scale target prioritization. By moving beyond isolated pairwise classification, PEARL provides a scalable computational framework for proteome-wide reverse screening and for uncovering new therapeutic opportunities for peptide-based drugs.

## Materials and methods

### Overview of PEARL

PEARL is a heterogeneous graph-based framework designed for protein target recommendation in peptide repositioning. Given known peptide–protein association data and a query peptide with a large candidate target space, PEARL aims to assign each candidate protein a recommendation score and generate a ranked list of plausible targets. As shown in Fig. 1, the overall workflow of PEARL comprises three major components: heterogeneous graph construction, relation-aware graph encoding, and recommendation-oriented score refinement.

**Figure 1:**
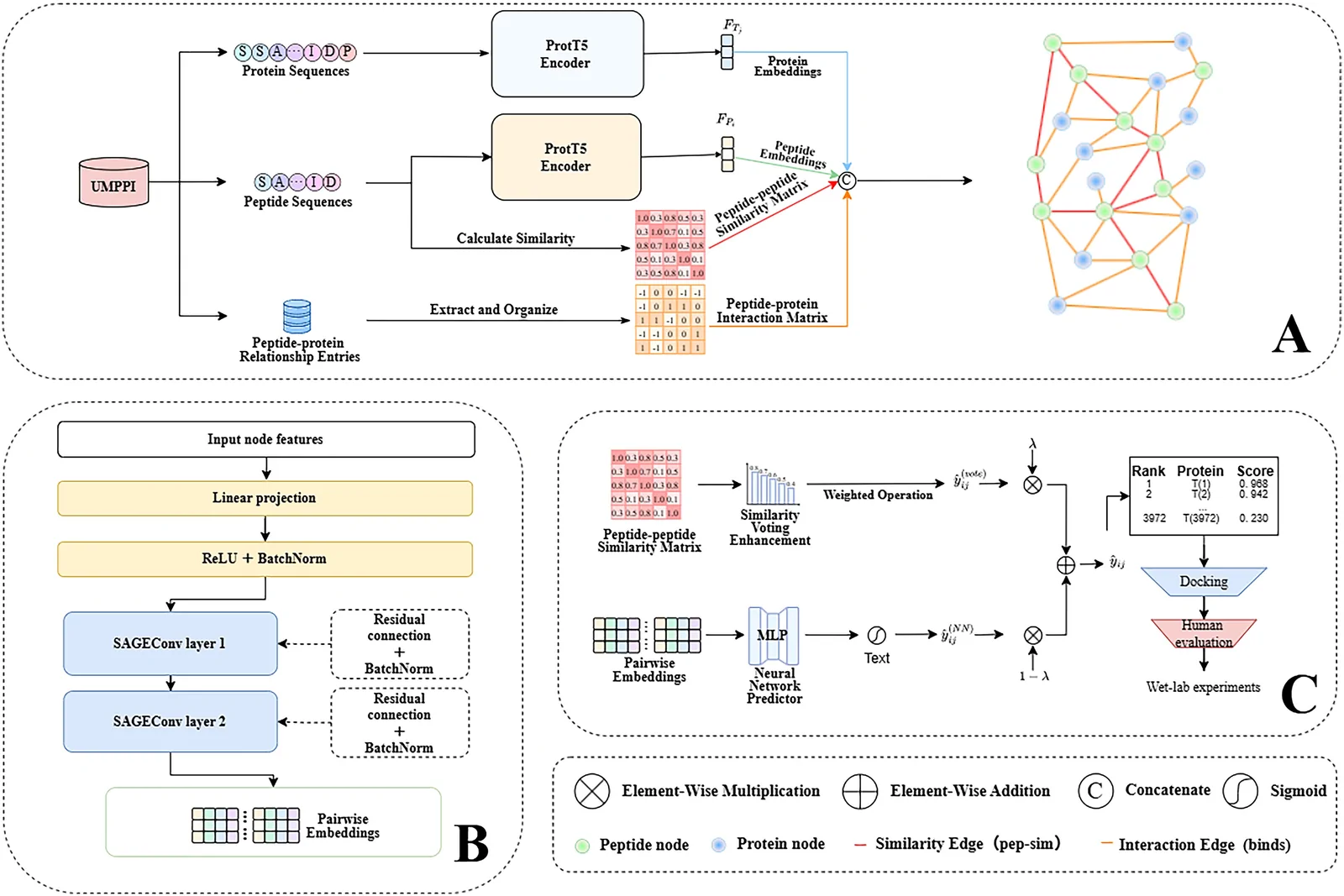
The overall workflow of PEARL; (A) peptide and protein sequences are encoded and assembled into a heterogeneous graph; (B) a graph encoder learns node embeddings via message passing; (C) calculate predicted interaction scores for targets and rank them, each score is computed through weighted aggregation of neural network prediction and voting enhancement based on peptide similarity.

Specifically, peptide and protein sequences are first transformed into dense representations using a pretrained ProtT5 encoder. Based on these sequence-derived features and known association data, PEARL constructs a heterogeneous graph in which peptides and protein targets are represented as distinct node types, while known peptide-protein associations and peptide–peptide similarity relationships are encoded as graph edges. A two-layer heterogeneous GraphSAGE encoder is then applied to propagate information across the graph and learn context-aware embeddings for both peptides and protein targets. For each candidate peptide-target pair, PEARL computes a base interaction score using a multilayer perceptron operating on multiple pairwise combinations of the learned embeddings. Finally, to better align prediction with the practical objective of target prioritization, PEARL further refines these scores by incorporating similarity-based evidence from related peptides, thereby producing a final ranked list of candidate protein targets. The following subsections describe each component in detail.

### Dataset

Our study employed the dataset of Unveiling Multilevel Protein–Peptide Interaction (UMPPI) [18] study for model training and test. The dataset comprises 17 978 peptide-protein pairs, including 8989 positive and 8989 negative sample pairs, involving a total of 6603 peptides and 3972 proteins.

To obtain the PepPI matrix and the peptide–peptide similarity matrix, we process the original dataset as follows: first, we construct the PepPI matrix based on peptide-protein relationship entries, where “1” indicates an interaction exists, “0” indicates no interaction exists, and “-1” indicates the relationship is unknown. Then, the current fusion model uses three feature: a ProtT5 cosine peptide-similarity matrix, an alignment-based peptide sequence-similarity matrix generated by normalized Smith-Waterman local alignment, and a training-fold-specific peptide interaction-profile similarity matrix reconstructed from positive training pairs only to prevent label leakage. Peptide and protein nodes are initialized with ProtT5 embeddings.

We evaluated the model using both random five-fold splitting and clustered inductive splitting. The clustered setting used similarity thresholds of 0.3, 0.4, and 0.5, and included the new_peptide_balanced, new_protein_balanced, and both_new_balanced protocols. In matched comparison experiments, train, validation, and test graphs were built independently within each split and used split-local nodes only.

### Sequence representation and multi-view peptide similarity

Both peptide and protein nodes are initialized with ProtT5 embeddings [19]. Residue-level embeddings are mean-pooled to sequence-level vectors and standardized separately for peptides and proteins before graph construction. For each peptide pair, PEARL computes three complementary similarity views: an embedding view given by the cosine similarity of the pooled ProtT5 vectors, a sequence view given by normalized Smith–Waterman local-alignment scores (BLOSUM62) [20], and an interaction view given by the Jaccard overlap of their binding profiles, where each profile records the protein targets a peptide binds among the training positives only. The three views are fused by a convex combination


$${S}_{ik}^{\mathrm{fuse}}={w}_{\mathrm{emb}}{S}_{ik}^{\mathrm{emb}}+{w}_{\mathrm{seq}}{S}_{ik}^{\mathrm{seq}}+{w}_{\mathrm{int}}{S}_{ik}^{\mathrm{int}},{w}_{\mathrm{emb}}+{w}_{\mathrm{seq}}+{w}_{\mathrm{int}}=1$$

Each view is min–max normalized to [0,1] before fusion, and self-similarities are set to zero. The fusion weights were selected by an ablation over view combinations on the random split, in which the three-view configuration achieved the best AUC, Area Under the Precision-Recall Curve (AUPR), and Matthews correlation coefficient (MCC) and was adopted as the main configuration (Supplementary Table S1). Its advantage over the embedding-only baseline holds across most cold-start splits and redundancy cutoffs, with the largest and most consistent gains in the protein cold-start (target-relocation) setting (Supplementary Table S2). Because the interaction view is derived from observed training positives, held-out peptides that never appear in the training positives receive all-zero interaction rows, so no target information leaks across the split; accordingly, the fused matrix is rebuilt within each fold. The exclusion of protein–protein edges is an evidence-based design choice rather than an omission (Supplementary Table S5).

### Graph construction and heterogeneous encoding

PEARL builds a heterogeneous graph with two node types, peptides and proteins, connected by observed peptide-protein binding edges and by peptide–peptide similarity edges. A peptide–peptide edge *p_i_, p_k_* is retained when the fused similarity exceeds a threshold alpha 0.5, with an additional top-K truncation that keeps only the strongest neighbors of each node:


$${E}_{pp}=\left\{\left({p}_i,{p}_k\right)\ |{S}_{ik}^{\mathrm{fuse}}>\alpha \right\}$$

In the main configuration, at most 20 peptide neighbors are retained per node. The framework also supports protein–protein similarity edges built from a precomputed protein similarity matrix; these are disabled in the target-relocation (protein cold-start) setting, which is the main configuration reported here, because held-out proteins have no reliable similarity neighbors in the training graph.

The graph is implemented as a Deep Graph Library (DGL) heterograph [21] and encoded with a two-layer heterogeneous GraphSAGE model [22]. The first layer maps node states from 512 to 256 dimensions with mean aggregation across relations; the second maps 256 to 128 dimensions with GCN-style GraphSAGE aggregation. Residual projections, batch normalization, and dropout are applied after each layer.

### Pair scoring, refinement, and optimization

For each candidate peptide-protein pair, the final peptide and protein embeddings are combined into a composite pair representation ${z}_{ij}$, formed by concatenating the two node embeddings, their element-wise product, their absolute difference and their squared difference. A multilayer perceptron then produces the base neural interaction score. To align prediction with cold-peptide recommendation, PEARL further refines the base score by transferring evidence from similar peptides. For a query peptide *i*, the voting neighborhood is defined from the fused similarity graph after thresholding, and at most five neighbors are used. Only neighbors with known labels for protein j contribute to the vote.


$${v}_{ij}=\frac{\sum_{k\in{N}_K(i),{y}_{kj}\ge 0}{S}_{ik}^{\mathrm{fuse}}{y}_{kj}}{\sum_{k\in{N}_K(i),{y}_{kj}\ge 0}{S}_{ik}^{\mathrm{fuse}}+\varepsilon },{s}_{ij}=\left(1-\lambda \right){s}_{ij}^{\mathrm{NN}}+\lambda{v}_{ij}$$

This refinement is applied during validation, testing, and inference, but not during back-propagation.


$$L=-\frac{1}{\mid{\Omega}_{\mathrm{train}}\mid }{\sum}_{\left(i,j\right)\in{\Omega}_{\mathrm{train}}}\left[{y}_{ij}\mathit{\log}{s}_{ij}^{\mathrm{NN}}+\left(1-{y}_{ij}\right)\ \mathit{\log}\left(1-{s}_{ij}^{\mathrm{NN}}\right)\right]$$

### Molecular dynamics simulation experiment procedure and operational details

The structure of the human SPSB2 (PDB ID: 9RV5) was retrieved from the RCSB Protein Data Bank, after which crystallographic waters, ions, and non-covalently bound heteroatoms were removed. Protonation states were assigned at pH 7.4, and the resulting structures were saved in RCSB Protein Data Bank (PDB) format for subsequent calculations. The CIGB-814 peptide was obtained by extracting its corresponding sequence from heat shock protein 60 (HSP60) and was subsequently subjected to structural preprocessing prior to model inference and docking analysis.

To characterize the interaction pattern between peptide candidates and their target proteins, molecular docking was performed using HADDOCK2.4 [23] with a predefined binding pocket. Molecular dynamics simulations were performed using Amber24 with the pmemd.cuda engine [24]. The ff19SB force field and simple point charge (SPC) water model were applied, and the SPSB2–CIGB814 peptide complexes were then solvated in a truncated octahedral box with a 14 Å buffer and neutralized with Na^+^ and Cl^−^ ions. A 2.0 fs time step was used, with bonds involving hydrogen atoms constrained using SHAKE, and long-range electrostatic interactions treated using particle mesh Ewald (PME) with a 9.0 Å cutoff. Following energy minimization and standard equilibration, production simulations were carried out for 100 ns under constant number of particles, pressure, and temperature (NPT) conditions at 300 K and 1 atm, and trajectories were saved every 10 ps for analysis.

### Surface plasmon resonance experiment procedure and operational details

Surface plasmon resonance (SPR) experiments were performed at 25°C using a Biacore T200 instrument (GE Healthcare). According to the manufacturer’s instructions, the recombinant human SPSB2 protein was immobilized on the surface of the CM5 sensor according to the activation, immobilization, and blocking standard protein amine coupling procedure. During the immobilization process, HEPES-buffered saline with surfactant P20 (HBS-P) + buffer (containing 0.05% Tween-20, 0.01 M HEPES pH 7.4, 0.15 M NaCl) was used as the running buffer. The binding affinity was measured at 25°C. The buffer contained 0.01 M 4-(2-hydroxyethyl)-1-piperazineethanesulfonic acid (HEPES) pH 7.4, 0.15 M NaCl, and 0.05% Tween-20. The analyte was determined by a two-fold serial dilution method. The concentration was 51.2 , 25.6, 12.8, 6.4, 3.2, 1.6, 0.8, 0.4 μM, injected at a flow rate of 30 μl/min for 100 s, followed by 100 s of dissociation time [25]. The SPR data for each analyte concentration were collected by Biacore T200 Evaluation Software (GE Healthcare). Five continuous concentrations were selected and fitted by a 1:1 binding kinetic model to calculate the binding rate (ka), dissociation rate (kd), and equilibrium dissociation constant (KD = kd / ka).

## Results and discussion

### Evaluation metrics

We first evaluated PEARL against five competitive baselines—UMPPI, CAMP, Peptriever, PepCA, and HGT-PepPI—using five-fold cross-validation on the benchmark dataset (Table 1). PEARL achieved the best performance across all three metrics, with a mean AUC of 0.868 ± 0.005, AUPR of 0.855 ± 0.011, and MCC of 0.571 ± 0.006. Compared with the strongest baseline, HGT-PepPI (AUC 0.824, AUPR 0.807, MCC 0.501), PEARL improved AUC by 4.4, AUPR by 4.8, and MCC by 7.0 percentage points, and it surpassed the remaining methods by even larger margins (AUC ≤ 0.791). The consistently small SDs across folds (≤0.011 for all metrics) indicate that these gains are stable rather than driven by fold-specific variance. Notably, PEARL delivered the largest relative improvement on MCC, a metric that jointly reflects performance on both positive and negative classes, demonstrating that the model not only ranks interactions well but also yields better-calibrated discrimination between binding and non-binding pairs.

**Table 1:** Results of performance comparison via five-fold cross-validation.

	AUC	AUPR	MCC
UMPPI	0.791 ± 0.009	0.767 ± 0.014	0.435 ± 0.022
CAMP	0.789 ± 0.012	0.735 ± 0.011	0.432 ± 0.030
Peptriever	0.781 ± 0.008	0.806 ± 0.008	0.175 ± 0.044
PepCA	0.750 ± 0.033	0.719 ± 0.019	0.365 ± 0.040
HGT-PepPI	0.824 ± 0.007	0.807 ± 0.010	0.501 ± 0.008
PEARL	0.868 ± 0.005	0.855 ± 0.011	0.571 ± 0.006

### Generalization under cold-start splits

To evaluate the effectiveness of PEARL, we compared it with four baseline methods, CAMP, PepCA, UMPPI, and HGT-PepPI, on the UMPPI benchmark. The evaluation was conducted under three scenarios, namely “Novel protein,” “Novel peptide,” and “Novel pair,” and model performance was assessed using the area under the receiver operating characteristic curve (AUC) and the MCC. The results across different clustering thresholds are summarized in Fig. 2. As shown in Fig. 2, PEARL achieved the best overall AUC performance across all three evaluation scenarios. In the “Novel protein” setting, PEARL consistently ranked first from the non-clustered split (NA) to the most stringent clustered split (0.5), indicating strong generalization to unseen proteins. A similar pattern was observed in the “Novel peptide” setting, where PEARL also yielded the highest AUC across all thresholds, although the margin over HGT-PepPI became smaller as the split became more stringent. In the “Novel pair” setting, which represents the most challenging scenario because both peptides and proteins are unseen during testing, PEARL still maintained the best AUC across all thresholds, suggesting superior robustness under severe distribution shift. The MCC results largely supported the same conclusion. In the “Novel protein” setting, PEARL consistently achieved the highest MCC at all thresholds. In the “Novel pair” setting, PEARL remained the best or among the best-performing methods across clustered splits. In the “Novel peptide” setting, HGT-PepPI showed slightly higher MCC than PEARL at some stricter thresholds, whereas PEARL still retained competitive MCC and the best AUC overall. Taken together, these results indicate that PEARL provides stronger discrimination ability and more robust cross-cluster generalization than existing baselines. Notably, the performance of all methods declined as the clustering threshold increased from NA to 0.5. This trend indicates that reducing sequence similarity between the training and test sets substantially increases the difficulty of binary interaction prediction. Among the three scenarios, the “Novel pair” setting consistently produced the lowest AUC and MCC values for all methods, confirming that simultaneous novelty in both peptides and proteins poses the greatest challenge. Nevertheless, PEARL showed the strongest overall robustness under these strict inductive evaluation settings.

**Figure 2:**
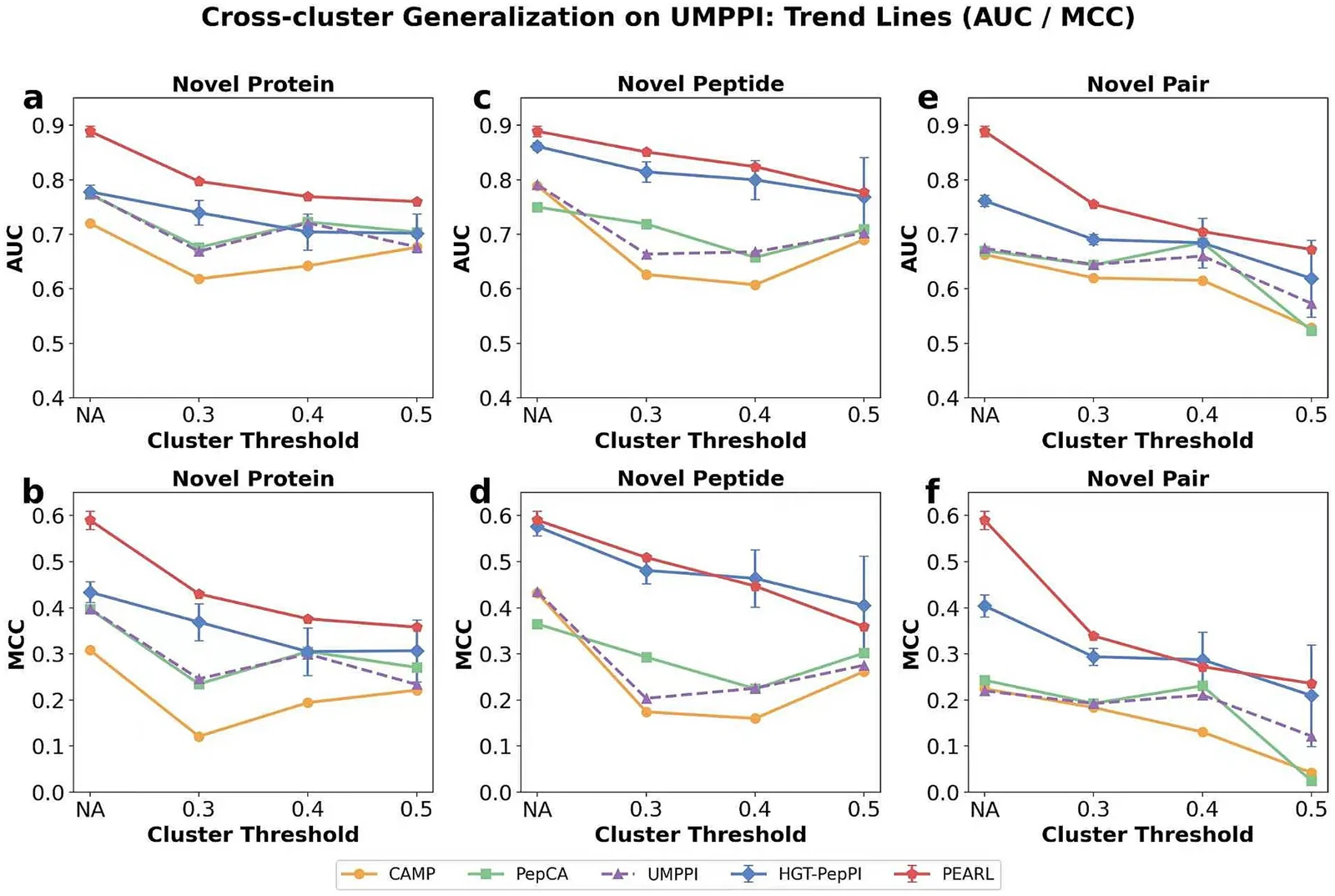
Cross-cluster binary interaction prediction performance of PEARL and baseline methods on the UMPPI benchmark; the performance of CAMP, PepCA, UMPPI, HGT-PepPI, and PEARL is shown under three evaluation scenarios: “Novel protein,” “Novel peptide,” and “Novel pair”; performance was assessed using AUC and MCC across four clustering conditions (NA, 0.3, 0.4, and 0.5); panels (a, c, and e) show the AUC results, and panels (b, d, and f) show the corresponding MCC results; NA indicates the non-clustered split.

### Independent test evaluation

#### Performance on independent test sets

We benchmarked PEARL against four representative PepPI predictors—HGT-PepPI, UMPPI, CAMP, and PepCA—on two independent test sets, Test167 and Test1440. To ensure a strictly fair comparison, all methods were trained on the same random five-fold split and evaluated under an identical graph-construction protocol, and predictions were ensembled over the five folds. As shown in Fig. 3, PEARL achieved the best or comparable performance across all metrics on both datasets. On Test167, PEARL attained an AUC of 0.863 and an AUPR of 0.850, both exceeding the strongest baseline HGT-PepPI (0.857 and 0.849) and substantially outperforming UMPPI, CAMP, and PepCA (AUC ≤ 0.57). On the larger Test1440 set, which contains a higher proportion of previously unseen peptides and proteins, PEARL again ranked first with an AUC of 0.871 and an AUPRof 0.833, surpassing HGT-PepPI (0.857 and 0.808) by 1.4 and 2.5 percentage points, respectively, while the remaining baselines degraded markedly (MCC ≤ 0.18). Because the MCC depends on a decision threshold and is sensitive to distribution shift in the independent sets, we regard the threshold-independent AUC and AUPR as the primary criteria; under both metrics PEARL is consistently the top performer. Notably, PEARL’s margin over the baselines is larger and more stable on the bigger, more challenging Test1440, indicating that its advantage arises from genuine generalization to unseen peptide–protein pairs rather than small-sample variance. This generalization advantage stems from PEARL’s multi-view similarity fusion, which enriches the peptide–protein heterogeneous graph beyond the limited set of known interactions.

**Figure 3:**
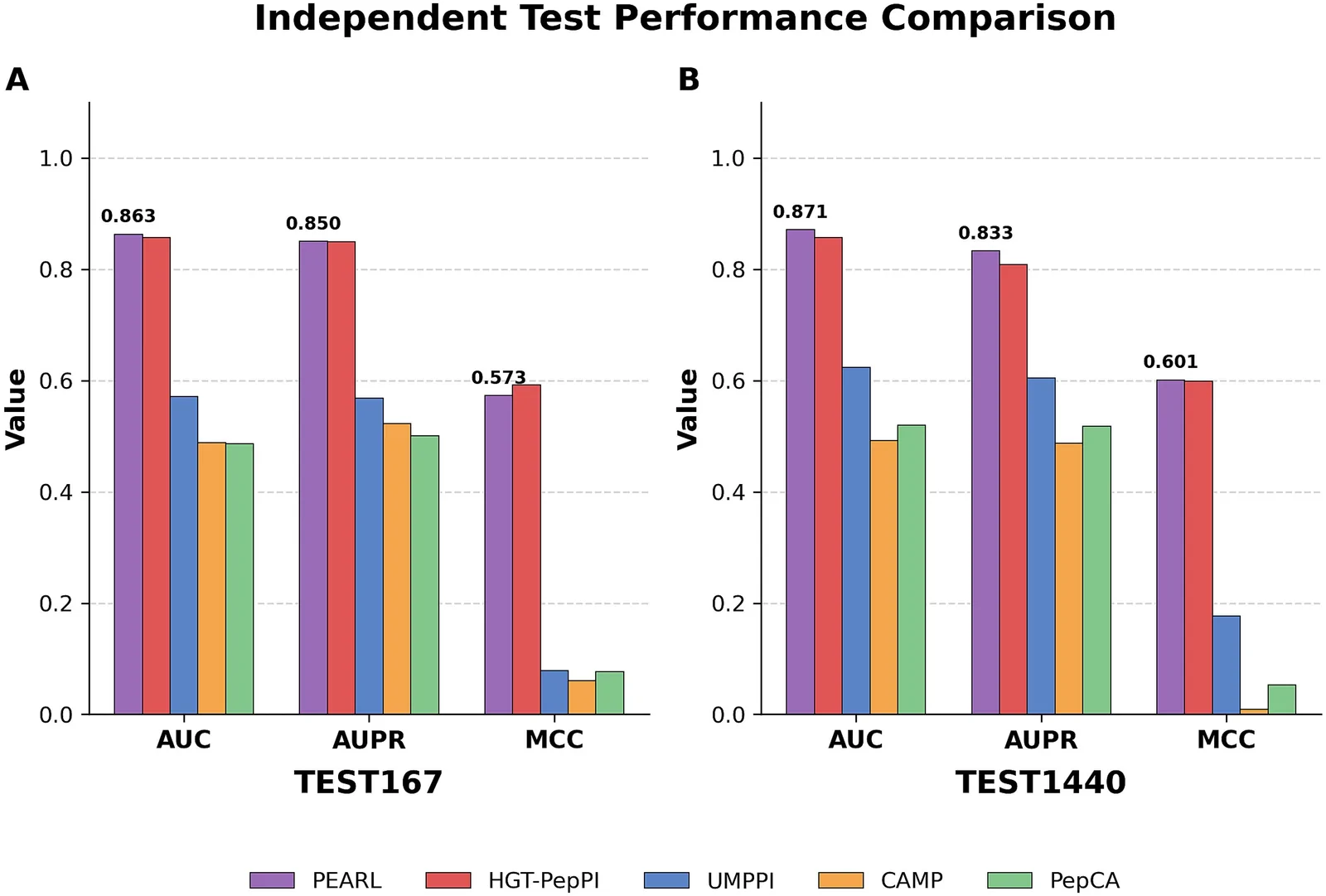
Performance comparison on independent test sets; bar plots of AUC, AUPR, and MCC for PEARL and four baseline methods (HGT-PepPI, UMPPI, CAMP, PepCA) on (A) Test167 and (B) Test1440.

### Hyperparameter sensitivity, calibration, and latent-space organization of PEARL

We examined model behavior under the novel-peptide cold-start setting by systematically varying the similarity threshold α and fusion weight λ (Supplementary Figure S3) over the full α × λ grid (α∈{0.3,0.5,0.7}, λ∈{0,0.25,0.5,0.75,0.9}; Fig. 4A). Test AUC was optimal around α = 0.5; when α was too small (0.3), weakly similar neighbors likely introduced noise, whereas larger values (0.7) produced an overly sparse similarity graph, in both cases reducing performance. Setting λ = 0, which disables similarity-guided fusion, yielded the lowest AUC and made performance nearly invariant to α (0.808–0.812), indicating that the benefit of α is expressed primarily through the similarity graph and neighbour-voting pathway. As λ increased, performance improved and then plateaued near λ ≈ 0.75, with the best result observed at α = 0.5, λ = 0.75 (AUC = 0.856). Relative to the fusion-off setting, this configuration improved AUC by ~0.05, and we therefore adopted α = 0.5 and λ = 0.75 as the default setting.

**Figure 4:**
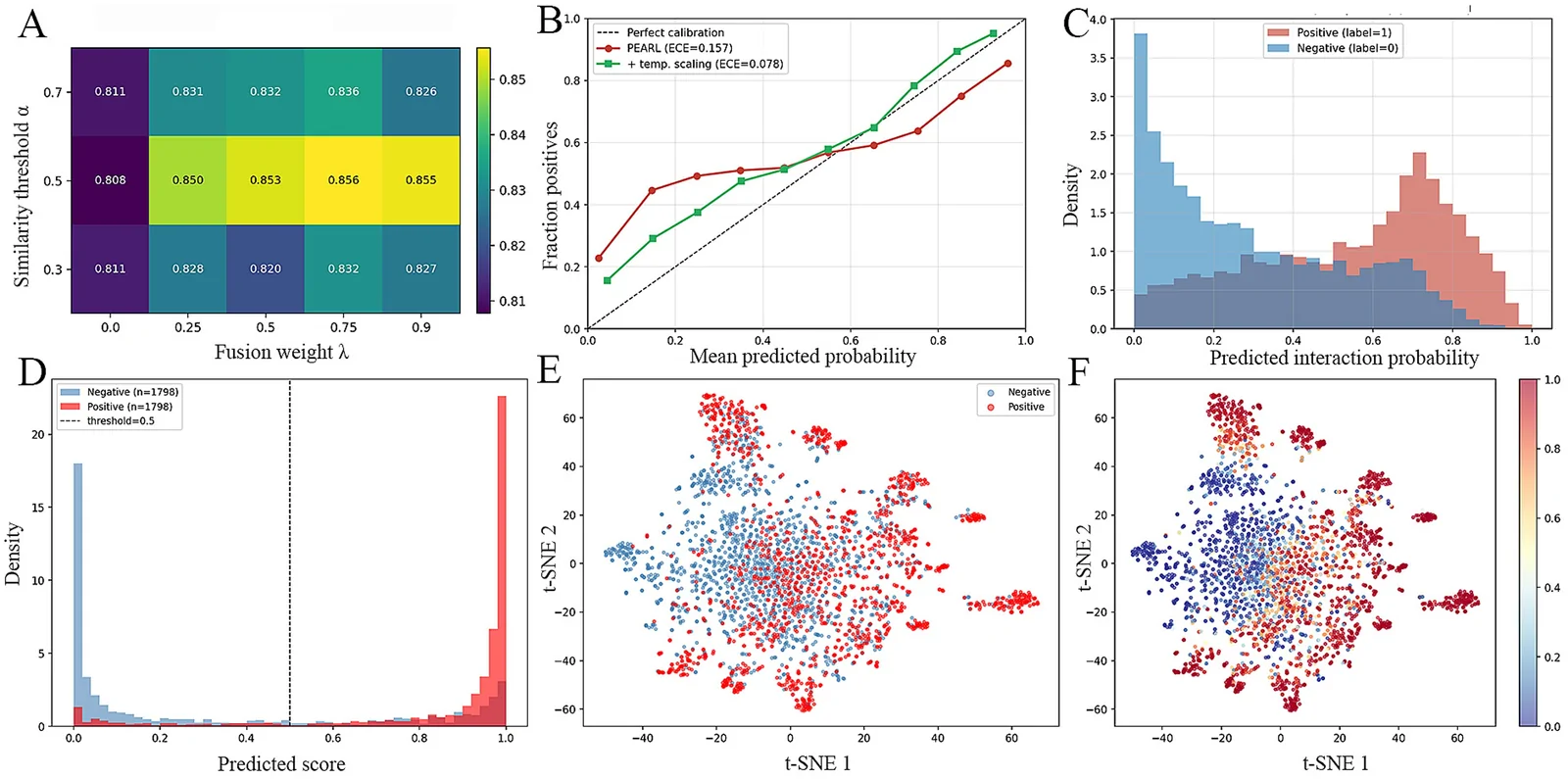
Hyperparameter sensitivity, calibration, and latent-space organization of PEARL under novel-peptide cold-start evaluation; (A) heat map of test AUC across the similarity threshold α and fusion weight λ; (B) fold-pooled reliability diagram before and after post hoc temperature scaling; (C) fold-pooled predicted-probability distributions for positive and negative pairs after calibration; (D) predicted-score distribution in a representative test fold; (E) t-SNE visualization of pair embeddings from the same fold, coloured by ground-truth label; (F) the same t-SNE embedding coloured by predicted score, showing a continuous score gradient across the latent manifold.

We then assessed whether PEARL produced well-calibrated probabilities. In the fold-pooled cold-start evaluation, the raw model outputs showed moderate miscalibration (ECE = 0.157, Brier score = 0.226; Fig. 4B). Post hoc temperature scaling substantially improved calibration, reducing expected calibration error (ECE) to 0.078 and the Brier score to 0.203, while preserving the overall separation between positive and negative pairs (Fig. 4B and C). Thus, although PEARL already ranked interacting pairs effectively, a simple recalibration step made its probability estimates more reliable for downstream interpretation.

To visualize how predictions were organized in the learned latent space, we further examined a representative test fold containing 3596 peptide–protein pairs (Fig. 4D–F). The score distribution was strongly bimodal, with negative pairs concentrated near zero and positive pairs shifted towards high scores, although overlap remained in the intermediate range (Fig. 4D). In the pair-embedding manifold, positive and negative examples occupied overlapping but structured regions rather than a single undifferentiated cloud (Fig. 4E). Coloring the same embedding by predicted score revealed a smooth transition from low- to high-confidence regions, with compact peripheral islands enriched for highly scored pairs and more mixed central regions corresponding to harder cases (Fig. 4F). Together, these analyses show that PEARL combines robust cold-start performance with a structured latent geometry and calibratable confidence estimates.

PEARL places functionally related targets into a shared latent neighborhood. To test whether this organization captured more than direct sequence homology, we analyzed the HIV-1 fusion peptide AVGIGAVF in the PEARL receptor-embedding space (Supplementary Figure S1). We computed cosine similarity between five immunoglobulin-related reference targets of AVGIGAVF and PEARL-ranked candidate proteins (Fig. 5). Importantly, the high- and low-scoring groups were defined by the final PEARL interaction score, whereas the heatmap color reports cosine similarity in receptor-embedding space; thus, a strictly monotonic correspondence between rank and color intensity is not expected. Instead, the relevant pattern was a group-level shift: high-scoring candidates were enriched for antigen-presentation and immune-recognition proteins, including MHC-related molecules, T-cell-receptor-associated chains, B2M, CD1D1, FCGRT, PROCR, and GZMH, whereas low-scoring candidates were dominated by nuclear receptors. On average, the high-scoring immune-recognition candidates were substantially more proximal to the reference antibody-related targets than the low-scoring nuclear-receptor candidates (mean cosine similarity, 0.51 versus −0.42), and this contrast remained evident after global z-score normalization. Pairwise global alignments across the five known immunoglobulin-related targets and the high-scoring immune-recognition predictions showed only modest direct sequence identity overall (mean known-versus-predicted identity, 26.8%; maximum, 34.1%), although several expected local similarity modules were evident within the major histocompatibility complex (MHC)/T-cell receptor (TCR)-related subset (Supplementary Fig. S2). Together, these results indicate that PEARL organizes AVGIGAVF targets by functional neighborhood rather than by simple sequence identity alone.

**Figure 5:**
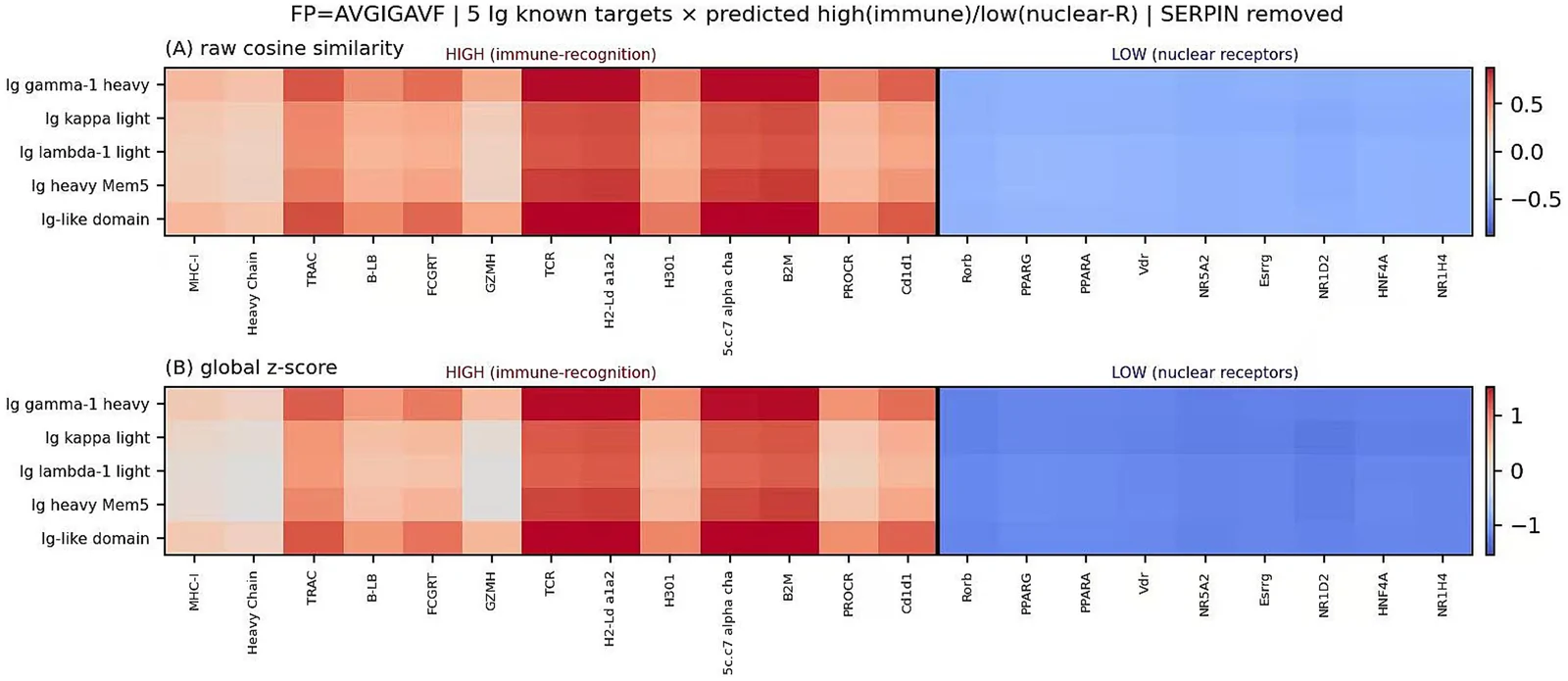
PEARL places immune-recognition-associated targets of AVGIGAVF in a shared latent neighborhood; (A) raw cosine similarity between five known immunoglobulin-related targets of the HIV-1 fusion peptide AVGIGAVF (rows) and selected high-scoring and low-scoring predicted protein targets (columns) in the PEARL receptor-embedding space; high-scoring predictions are enriched for immune-recognition-associated proteins, whereas low-scoring predictions are enriched for nuclear receptors; (B) the same matrix shown after global *z*-score normalization, confirming that the separation is not driven by a small number of extreme values.

### Case study on CIGB-814

To evaluate PEARL’s practical applicability on peptide drug repurposing, we employed it to recommend potential targets for the immunomodulatory peptide drug CIGB-814, which is absent from the UMPPI dataset. CIGB-814 is a 27-mer altered peptide ligand derived from a CD4+ T-cell epitope of the human HSP60, an autoantigen implicated in the pathogenesis of rheumatoid arthritis (RA) [26]. Previous studies have demonstrated its therapeutic potential in RA treatment [27, 28].

The top 15 candidate targets predicted by PEARL are listed in Supplementary Table S3. Among them, SPRY domain-containing SOCS box protein 2 (SPSB2) was ranked first with a predicted interaction score of ~0.94, substantially higher than the second-ranked target, TNF receptor-associated factor 6, which received a score of ~0.74. Based on this ranking result, SPSB2 was selected for further structural and experimental investigation.

To explore whether the top-ranked prediction was structurally plausible, molecular docking was performed using HADDOCK. The resulting docking model positioned CIGB-814 within a surface-exposed groove of SPSB2, suggesting a potential binding interface with favorable geometric complementarity between the peptide and the protein surface (Fig. 6A). In addition, four low-ranked proteins predicted by PEARL were subjected to the same docking protocol as negative controls. Although docking clusters could be generated for these proteins, they exhibited substantially less favorable HADDOCK scores and markedly higher restraint violation energies than SPSB2 (Supplementary Table S4), indicating poorer structural compatibility with CIGB-814.

**Figure 6:**
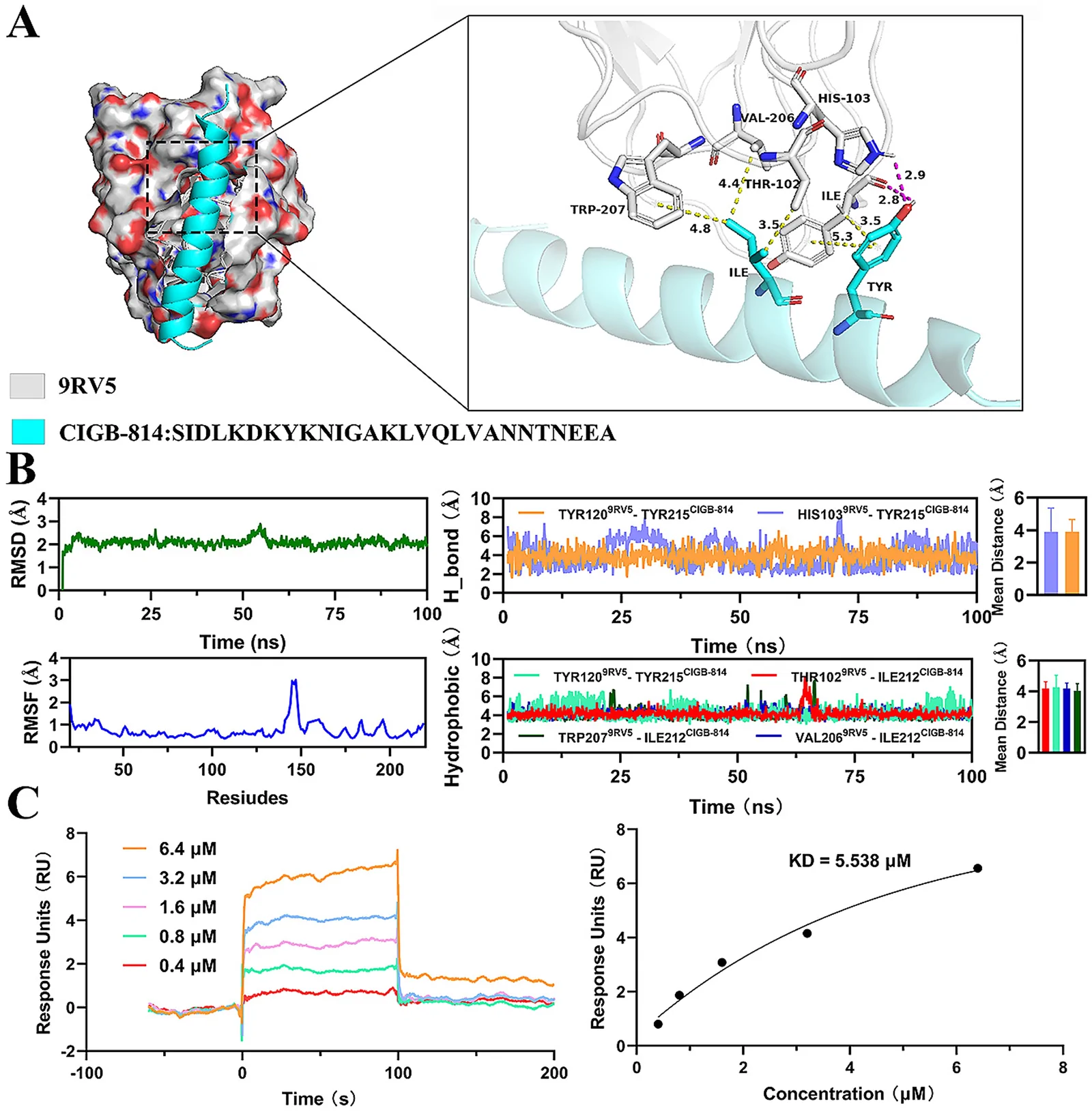
Structural and experimental validation of the interaction between CIGB-814 and SPSB2; (A) HADDOCK docking model of the CIGB-814-SPSB2 complex; (B) MD simulation analysis of the CIGB-814-SPSB2 complex, including RMSD, RMSF, and representative intermolecular interactions; (C) SPR analysis of the interaction between CIGB-814 and SPSB2.

To further evaluate whether this predicted binding mode could be maintained under dynamic conditions, a 100-ns MD simulation was performed. The backbone RMSD rapidly converged and remained relatively stable throughout the simulation, fluctuating around ~2 Å, indicating that the docked complex maintained an overall stable conformation (Fig. 6B). Simultaneously, hydrogen bond interactions involving Tyr120 and His103, together with hydrophobic contacts associated with Thr102, Tyr120, Val206, and Trp207, were dynamically maintained during the simulation. Notably, these interface residues also exhibited relatively low atomic fluctuations in the root mean square fluctuation (RMSF) profile, further supporting their contribution to peptide binding and complex stabilization.

It should be noted that molecular docking and molecular dynamics simulations primarily provide structural and physicochemical support for a predicted interaction and do not, by themselves, establish biological relevance. Therefore, experimental validation was further performed using SPR. The sensorgrams revealed a clear concentration-dependent binding response between CIGB-814 and SPSB2 (Fig. 6C). Steady-state fitting yielded an apparent dissociation constant (KD) of 5.538 μM, indicating a measurable micromolar binding affinity between the peptide and the protein.

These data support SPSB2 as an experimentally supported binding partner of CIGB-814 and nominate it for future functional investigation. They do not, by themselves, establish therapeutic relevance.

### Discussion

Our results show that reformulating peptide target discovery as a recommendation problem is advantageous over treating PepPIs as isolated binary classification events. By combining multi-view peptide similarity with heterogeneous graph learning, PEARL can propagate informative context across related peptides while preserving the PepPI structure needed for target ranking. This design likely explains why PEARL showed the clearest gains not only in standard cross-validation but also in clustered cold- start and independent test settings, where simple memorization of highly similar training examples is less effective. The performance pattern further suggests that PEARL captures biologically meaningful relational signals rather than relying only on sequence similarity. Its advantage was most consistent in AUC and AUPR, as well as in MCC for the novel-protein and novel-pair settings, indicating strong ranking ability and robust discrimination under distribution shift. The latent-space analysis of AVGIGAVF supports this interpretation, as high-scoring candidates were enriched for immune-recognition-associated proteins despite only modest direct sequence identity to known reference targets. The improvement after temperature scaling further indicates that part of the remaining limitation lies in probability calibration rather than in representation quality alone. Several limitations define the current scope of PEARL. First, the framework relies mainly on peptide–protein associations and peptide-side similarity, whereas protein-side relational information and explicit structural interaction features are only partially represented. Second, although PEARL performed strongly on the current benchmark and independent test sets, its behavior in larger and noisier proteome-scale settings remains to be established. Third, the CIGB-814-SPSB2 study provides encouraging structural and biophysical support, but a single experimentally validated case is not sufficient to establish broad translational utility. Future work should therefore focus on incorporating richer protein-side priors, improving calibration and decision robustness under severe cold-start conditions, and expanding experimental validation across more therapeutic peptides and target classes.

## Conclusion

In summary, PEARL addresses peptide drug repurposing as a protein target recommendation problem by combining heterogeneous graph learning, multi-view peptide similarity fusion, and similarity-aware score refinement. Across five-fold cross-validation, clustered cold-start evaluation, and independent test sets, PEARL consistently showed stronger or more stable performance than existing baseline methods. The case study on CIGB-814 further demonstrated that PEARL can generate experimentally supported target hypotheses, with SPSB2 identified as a previously unrecognized binding partner and validated by SPR. These findings suggest that PEARL provides a practical computational framework for large-scale peptide target prioritization, especially in settings where unseen peptides or proteins are common. A current limitation is that the model still depends on the coverage and quality of available PepPI data and uses relatively limited protein-side relational context. Extending PEARL with richer protein representations and broader experimental validation will be an important next step toward translational application in peptide drug repurposing.

**Key Points**

- PEARL reframes peptide drug repurposing as a protein target recommendation problem rather than a standalone peptide-protein pair classification task.
- PEARL combines multi-view peptide similarity, peptide-protein associations, and heterogeneous graph learning to generate context-aware target rankings.
- PEARL outperforms current baselines in five-fold cross-validation, clustered cold-start evaluation, and independent test sets, demonstrating robust generalization to unseen peptides and proteins.
- PEARL identified SPSB2 as a promising target for the unseen therapeutic peptide CIGB-814, supported by molecular docking, molecular dynamics simulations, and experimental binding validation.

## Supplementary Material

Supporting_Information_bbag551(1)

## Data Availability

The source code of PEARL is available on GitHub (https://github.com/lennyliu2000/PEARL). The data files used in PEARL are available in the Figshare (https://doi.org/10.6084/m9.figshare.31220953).
