# Supplementary material for: PEARL: novel protein targets recommendation model via heterogeneous graph representation learning
Source: Brief Bioinform. 2026 Oct 8;27(5):bbag551. doi: 10.1093/bib/bbag551 (PMC13647286; doi:10.1093/bib/bbag551)
Supplement: Supplementary file 1 [file supporting_information_bbag551(1).docx]

**Supporting Information**

**PEARL: Novel Protein Targets Recommendation Model via Heterogeneous Graph representation Learning**

Zixuan Liu^1,†^, Jiasi Luan^1,†^, Zhen Zhang^1^, Kui Tang^1^, Xingyu Qin^2^, Zhixiao Qi^1^, Borui Zhang^1^, Jun Liao^1,*^

1. School of Science, China Pharmaceutical University, Nanjing 211198, China

2. New Drug Screening and Pharmacodynamics Evaluation Center, State Key Laboratory of Natural Medicines, China Pharmaceutical University, Nanjing, 210009, China

^†^Zixuan Liu and Jiasi Luan are Co-first authors.

^*^ Corresponding Author: Jun Liao; Email: liaojun@cpu.edu.cn

**Supplementary Note 1:**

Table S1: View-combination ablation on the random split

| Configuration | View weights (emb/seq/int) | AUC | AUPR | MCC |
| --- | --- | --- | --- | --- |
| protT5 embedding only | 1.0 / –/ – | 0.8803 | 0.8745 | 0.5863 |
| embedding + sequence (BLAST) | 0.5 / 0.5 / – | 0.8760 | 0.8725 | 0.5875 |
| embedding + sequence (Morgan) | 0.5 / 0.5 / – | 0.8704 | 0.8661 | 0.5799 |
| embedding + sequence (Morgan) + interaction | 0.5 / 0.3 / 0.2 | 0.8725 | 0.8650 | 0.5616 |
| embedding + sequence (BLAST) + interaction | 0.5 / 0.3 / 0.2 | 0.8831 | 0.8768 | 0.5907 |

Table S2: Effect of multi-view similarity fusion under sequence-deduplicated cold-start splits.

| split_type | redundancy_cutoff | emb_only_AUC | fused_AUC | emb_only_AUPR | fused_AUPR | emb_only_MCC | fused_MCC |
| --- | --- | --- | --- | --- | --- | --- | --- |
| new_peptide | clu0.3 | 0.839 | 0.851 | 0.831 | 0.844 | 0.486 | 0.509 |
| new_peptide | clu0.4 | 0.816 | 0.824 | 0.811 | 0.821 | 0.407 | 0.447 |
| new_peptide | clu0.5 | 0.769 | 0.777 | 0.771 | 0.78 | 0.364 | 0.359 |
| new_protein | clu0.3 | 0.789 | 0.797 | 0.775 | 0.794 | 0.402 | 0.43 |
| new_protein | clu0.4 | 0.754 | 0.769 | 0.753 | 0.769 | 0.352 | 0.376 |
| new_protein | clu0.5 | 0.748 | 0.76 | 0.744 | 0.76 | 0.352 | 0.358 |
| both_new | clu0.3 | 0.74 | 0.755 | 0.732 | 0.746 | 0.321 | 0.339 |
| both_new | clu0.4 | 0.691 | 0.705 | 0.69 | 0.705 | 0.252 | 0.272 |
| both_new | clu0.5 | 0.676 | 0.672 | 0.682 | 0.671 | 0.249 | 0.236 |

**Supplementary Note 2:**

**Supplementary Fig. 1 |** Sequence-identity relationships among known and high-scoring immune-recognition-associated targets of AVGIGAVF. Heat map showing pairwise global-alignment percent identity among the five known immunoglobulin-related targets of AVGIGAVF and the high-scoring immune-recognition-associated predicted targets, ordered by hierarchical clustering.

**Supplementary Note 3:**

**Supplementary Fig. 2** | Joint effect of the similarity threshold α and fusion weight λ on PEARL.

**Supplementary Fig. 3 |** Sensitivity to the fusion weight λ and the λ = 0 ablation.

**Supplementary Note 4:**

Table S3: Target prediction ranking list for CIGB-814 through PEARL

| Rank | Score | UniProt ID | Target Name |
| --- | --- | --- | --- |
| 1 | 0.940835 | Q99619 | SPRY domain-containing SOCS box protein 2 |
| 2 | 0.735961 | Q9Y4K3 | TNF receptor-associated factor 6 |
| 3 | 0.717365 | P70365 | Nuclear receptor coactivator 1 |
| 4 | 0.713120 | Q5RAH9 | Baculoviral IAP repeat-containing protein 5 |
| 5 | 0.695000 | P25054 | Adenomatous polyposis coli protein |
| 6 | 0.670076 | P63104 | 14-3-3 protein zeta/delta |
| 7 | 0.667535 | P0AEX9 | Maltose/maltodextrin-binding periplasmic protein |
| 8 | 0.667307 | P20248 | Cyclin-A2 |
| 9 | 0.642449 | P18031 | Tyrosine-protein phosphatase non-receptor type 1 |
| 10 | 0.639915 | P0DOX5 | Immunoglobulin gamma-1 heavy chain |
| 11 | 0.637603 | D0VWR1 | Photosystem II CP47 reaction center protein |
| 12 | 0.636996 | Q92793 | CREB-binding protein |
| 13 | 0.632394 | O96013 | Serine/threonine-protein kinase PAK 4 |
| 14 | 0.628081 | P49951 | Clathrin heavy chain 1 |
| 15 | 0.612342 | P10898 | Photosystem II CP43 reaction center protein |

**Supplementary Note 5:**

Table S4: HADDOCK docking results for low-scoring protein targets and CIGB-814

| Parameters | 7EQX-27 | 7EQX-9 (Cluster3) | 2K89-27 (Cluster4) | 2K89-9 (Cluster2) |
| --- | --- | --- | --- | --- |
| HADDOCK score | 201.6±8.1 | 136.0±6.6 | 15.7±16.7 | 17.5±6.2 |
| Cluster size | 4 | 11 | 6 | 15 |
| RMSD (Å) | 10.3±0.1 | 1.3±0.1 | 4.4±1.1 | 5.5±0.3 |
| Van der Waals energy | −44.8±7.0 | −41.7±3.5 | −29.1±9.1 | −37.3±1.4 |
| Electrostatic energy | −134.0±7.2 | −242.3±9.2 | −271.4±51.9 | −180.0±46.9 |
| Desolvation energy | 3.0±0.6 | −6.4±1.2 | 7.3±2.5 | −7.3±3.5 |
| Restraints violation energy | 2701.5±48.4 | 2325.5±49.9 | 917.5±102.3 | 981.0±55.6 |
| BSA(Å²) | 1787.5±74.6 | 1319.9±17.0 | 1688.3±95.4 | 1099.5±52.2 |
| Z-Score | -1.9 | -1.6 | -1.7 | −1.9 |

**Supplementary Note 6:**

Table S5: Effect of multi-view similarity fusion under sequence-deduplicated cold-start splits.

| Setting | With prot-prot edges | without |
| --- | --- | --- |
| Random split | 0.852 | 0.868 |
| new_protein (clu 0.3) | 0.768 | 0.774 |
| new_protein (clu 0.4) | 0.721 | 0.745 |
| new_protein (clu 0.5) | 0.728 | 0.737 |
